# Comparative Genomic and Transcriptomic Analyses of LNCaP and C4-2B Prostate Cancer Cell Lines

**DOI:** 10.1371/journal.pone.0090002

**Published:** 2014-02-28

**Authors:** Lien Spans, Christine Helsen, Liesbeth Clinckemalie, Thomas Van den Broeck, Stefan Prekovic, Steven Joniau, Evelyne Lerut, Frank Claessens

**Affiliations:** 1 Laboratory of Molecular Endocrinology, Department of Cellular and Molecular Medicine, University of Leuven, Campus Gasthuisberg, Leuven, Belgium; 2 Urology, Department of Development and Regeneration, University Hospitals Leuven, Campus Gasthuisberg, Leuven, Belgium; 3 Translational Cell & Tissue Research, Department of Imaging and Pathology, University Hospitals Leuven, Leuven, Belgium; Florida International University, United States of America

## Abstract

The LNCaP and C4-2B cell lines form an excellent preclinical model to study the development of metastatic castration-resistant prostate cancer, since C4-2B cells were derived from a bone metastasis that grew in nude mice after inoculation with the LNCaP-derived, castration-resistant C4-2 cells. Exome sequencing detected 2188 and 3840 mutations in LNCaP and C4-2B cells, respectively, of which 1784 were found in both cell lines. Surprisingly, the parental LNCaP cells have over 400 mutations that were not found in the C4-2B genome. More than half of the mutations found in the exomes were confirmed by analyzing the RNA-seq data, and we observed that the expressed genes are more prone to mutations than non-expressed genes. The transcriptomes also revealed that 457 genes show increased expression and 246 genes show decreased expression in C4-2B compared to LNCaP cells. By combining the list of C4-2B-specific mutations with the list of differentially expressed genes, we detected important changes in the focal adhesion and ECM-receptor interaction pathways. Integration of these pathways converges on the myosin light chain kinase gene (MLCK) which might contribute to the metastatic potential of C4-2B cells. In conclusion, we provide extensive databases for mutated genes and differentially expressed genes in the LNCaP and C4-2B prostate cancer cell lines. These can be useful for other researchers using these cell models.

## Introduction

Prostate cancer (PCa) is the most frequently diagnosed cancer and third leading cause of death amongst men in Europe [1]. Despite its prevalence, a majority of men is diagnosed with localized, low-risk PCa and would never die because of their cancer when left untreated [2]. However, patients with high-risk and especially metastatic disease have a much higher risk of dying from PCa with reported PCa-specific mortality rates up to 28.8% for high-risk disease and 66.1% for metastatic disease at 10-years follow-up [3]. Recent epidemiological data have shown that almost 10% of all PCa patients are metastatic at the time of diagnosis, underlining the clinical importance of developing a better insight in the underlying mechanisms of metastatic PCa [4]. The genomic and transcriptomic changes that accompany the transformation of localized disease to metastatic castration-resistant PCa are being discovered, but are obstructed by the difficulties to obtain biopsies from the different stages of the disease [5], [6].

As an alternative, cell lines can be used as models to study the transition to metastatic castration-resistant PCa [7]. One of the best studied PCa cell lines undoubtedly is the LNCaP cell line. This cell line was derived from a needle biopsy taken from the left supraclavicular lymph node of a 50-year old Caucasian male [8]. This patient suffered from a rapidly progressing PCa with minimal and brief response to hormonal therapy and no response to chemotherapy. Subsequently, the C4-2 subline was derived from a tumor that developed in castrated nude mice injected with LNCaP cells. Finally, the C4-2B cell line was derived from a bone metastasis after orthotopic transplantation of C4-2 cells in nude mice [9], [10]. In other words, C4-2B is a metastatic derivative of the LNCaP cells. The LNCaP and C4-2B progression model therefore mimics the disease advancing from poorly tumorigenic, androgen-sensitive and non-metastatic in LNCaP, to metastatic and androgen-insensitive (or castration-resistant) in C4-2B.

For these two cell lines, changes in karyotype and genomic copy numbers, some point mutations, insertions and deletions have been described, but the comparison of the exome sequences have not been reported yet [9], [11]. The first goal of this study was therefore to obtain comprehensive exome data for LNCaP and C4-2B cells. Of course, a comparison of these mutational landscapes only makes sense in the presence of information on the activity of the affected genes. The latter was obtained from transcriptome analyses.

A first step to catalogue point mutations, insertions and deletions in the LNCaP cells was reported in Spans *et al.* [12]. Here, we report on a comparative whole exome and transcriptome sequencing study of both LNCaP and C4-2B cell lines. To our knowledge, this is the first direct and thorough comparison of this kind. Moreover, these databases can be very informative for preclinical studies for which both LNCaP and C4-2B cells are being used. They can also be used to generate hypotheses on the metastatic process, as exemplified for the MLCK pathway.

## Materials and Methods

### DNA isolation

The LNCaP cell line was obtained from the American Type Culture Collection, while the C4-2B cells were a kind gift from Dr. M. Stallcup (Norris Comprehensive Cancer Center, University of Southern California, USA) [9]. Both cell lines were grown in Roswell Park Memorial Institute medium (RPMI, Gibco, Invitrogen), containing 2 g/L glucose supplemented with 10% heat-inactivated fetal calf serum (FCS). The passage number of the LNCaP and C4-2B cells was 48 and 42 respectively. High-molecular weight DNA was extracted from cultured cells using the GenElute Mammalian Genomic DNA Miniprep kit (Sigma-Aldrich). After purification using ethanol precipitation with ammonium acetate, the concentration was quantified using a Nanodrop ND-1000 spectrophotometer (Thermo Fisher Scientific) and BioAnalyzer (Agilent).

### Whole exome sequencing

Whole-exome capture of the LNCaP cells was performed using the SureSelect Human All Exon System (Agilent) according to the manufacturer's instructions. Paired-end, 100 bp long sequencing reads were generated using the GAIIx sequencer (Illumina). The exome capture of the C4-2B cells was performed using the SeqCap EZ Exome version 2 kit (Roche Nimblegen) and paired-end 100 bp long reads were generated using the HiSeq2000 (Illumina).

Quality control was performed using FastQC software (version 0.10.1) (http://www.bioinformatics.bbsrc.ac.uk/projects/fastqc/) and Picard (version 1.22) (http://picard.sourceforge.net/). Sequencing reads were aligned to the human reference genome (hg19, NCBI Build 37) using BWA, where reads were trimmed when the quality was below 15 (version 0.5.9) [13]. Alignment files were processed further with Genome Analysis Toolkit (GATK) before variant calling and included duplicate removal, local realignment around known indels and base quality recalibration (version 1.0.5777) [14]. The samples were loaded individually to the GATK UnifiedGenotyper software. Point mutations and expression data were plotted using the Circos software (version 0.52) [15]. Comparison of point mutations was performed using Venny (http://bioinfogp.cnb.csic.es/tools/venny/index.html).

### RNA isolation

LNCaP and C4-2B cells, with passage numbers of 30 and 43 respectively, were plated in 6-well plates (1.75 million cells/well) and treated overnight (18 h) with 1 nM R1881 (Perkin Elmer). The cells were collected and washed with PBS. The cell pellet was used to extract total RNA using the RNeasy Mini Kit from Qiagen. The quality and purity of the RNA was inspected on a Nanodrop ND-1000 Spectrophotometer. The integrity of the RNA was verified on the BioAnalyzer at the Genomics Core of UZ Leuven.

### RNA sequencing

After selection of polyA+ RNA, the RNA was converted into cDNA libraries using the TruSeq RNA Sample Preparation kit (Illumina). After sequencing paired-end short reads of 100 bp with the HiSeq2000 (Illumina), normalized gene counts (Fragments Per Kilobase per Million of mapped reads, FPKM) were calculated via the Tuxedo pipeline (Tophat – Cufflinks – CummeRbund) [16]. In short, the RNA-seq data were aligned to the reference genome using TopHat (version 2.0.6) that utilizes Bowtie as the algorithmic core. The Cufflinks package (version 2.0.2) assembled the transcripts and detected differentially expressed genes and transcripts. CummeRbund (version 2.0.0) was used to visualize the gene expression data. Variant calling using the RNA-seq data was performed with GATK (version 2.2), after alignment with Tophat [14]. RNA-seq for both cell lines was performed in triplicate, allowing the identification of differentially expressed genes. For variant calling, the triplicates were aggregated to obtain higher coverage. Pathway-Express was used to determine, from a list of genes, whether in a specific pathway more genes are involved than would be expected by chance [17].

### Quantitative RT-PCR

cDNA was generated from RNA (1 µg) with Random Hexamer primers and RevertAid Reverse Transcriptase (Thermo Scientific). Quantitative Real Time PCR was performed using Platinum SYBR Green QPCR Supermix-UDG (Invitrogen). Results were normalized to the housekeeping gene β-actin and each sample was analyzed in triplicate. The sequence of the primers used are: β-actin forward 5′-ACCCAAGGCCAACCG-3′ and reverse 5′-TGTACGTTGCTATCCAGGCTGT-3′, TMPRSS2 forward 5′-CCTGCATCAACCCCTCTAACTG-3′ and reverse 5′-AGGCGAACACACCGATTCTC-3′, IGF1 forward 5′-TGGATGCTCTTCAGTTCGTG-3′ and reverse 5′-TCATCCACGATGCCTGTCT-3′, IGF1R forward 5′-GTACAACTACCGCTGCTGGA-3′ and reverse 5′-TGGCAGCACTCATTGTTCTC-3′.

### Accession numbers

Binary sequence alignment/map (BAM) files from whole exome sequencing data as well as RNA-seq data were deposited in the database of the European Nucleotide Archive with accession number PRJEB4877 and are accessible via http://www.ebi.ac.uk/ena/data/view/PRJEB4877. The sample accession numbers are ERS363578 and ERS363580 for whole exome sequencing data of LNCaP and C4-2B respectively. For the RNA-sequencing, the sample accession numbers are ERS363579 and ERS363581 for LNCaP and C4-2B cells respectively.

### Confirmation of non-synonymous variants

Variants of interest were confirmed by Sanger sequencing of amplified PCR products. Primers specific to the region containing the variant to be tested were designed using the NCBI Primer-Blast (http://ncbi.nlm.nih.gov/tools/primer-blast/) and obtained from Integrated DNA Technologies. Polymerase chain reactions were performed following standard protocols using Taq DNA polymerase (Thermo Scientific). Amplification of specific PCR fragments was confirmed by agarose gel electrophoresis. Sanger sequencing was performed at LGC Genomics. Sequence trace files were analyzed using Chromas Lite.

## Results

### Detecting point mutations with whole exome sequencing

We performed a whole-exome re-sequencing study for both LNCaP and C4-2B cells using 100 base pair, paired-end reads on the Illumina platform. This generated 49 and 80 million reads for LNCaP and C4-2B respectively (Table S1); for LNCaP cells, 74% of the exome was covered at least 20x, versus 88% for C4-2B cells. Sequencing characteristics and quality control data are similar for both datasets (Table S1 and Figure S1).

The point mutations in the exomes were detected using the GATK pipeline to which additional filtering was applied: only mutations which had at least 12× coverage and a mutation frequency above 30% were taken into account. Data were also filtered for absence of the base pair change in dbSNP130. Furthermore, strand bias was eliminated manually (Table S2). This resulted in lists of 2188 and 3840 non-synonymous point mutations in LNCaP and C4-2B cells, respectively (Table S3–4). Only 1784 mutations were common between both cell lines, clearly indicating the accumulation of more than 2000 additional mutations in the C4-2B genome. This large difference in mutation load cannot be explained by the slightly lower coverage of the LNCaP exome. Most likely, these additional C4-2B mutations have arisen during tumor progression and bone metastasis.

### Detecting point mutations in transcriptome sequencing

Transcriptome sequencing was performed initially to determine differential gene expression. RNA was isolated from LNCaP and C4-2B cells that had been treated for 18 hours with the synthetic androgen methyltrienolone. We obtained 157 and 131 million 100 base pair, paired-end reads for LNCaP and C4-2B cells. In these reads, the percentage of ribosomal, intronic and intergenic bases was very low (1.6% in total), resulting in a high coverage of mRNA bases (Table S1). As a measure for the quality of the transcriptome data, the variation in coverage along each transcript is shown in Figure S2. This shows no 5′ or 3′ bias although there is a somewhat lower coverage near the ends of the transcripts.

The workflow for the detection and subsequent filtering of point mutations was similar to that used for the exome sequencing described higher. We found 1505 and 1882 mutations in LNCaP and C4-2B cells respectively, of which 1054 were detected in both cell lines (Table S5); 451 were specific for LNCaP and 828 for C4-2B.

### Comparing exome with transcriptome sequencing data

**Figure 1:**
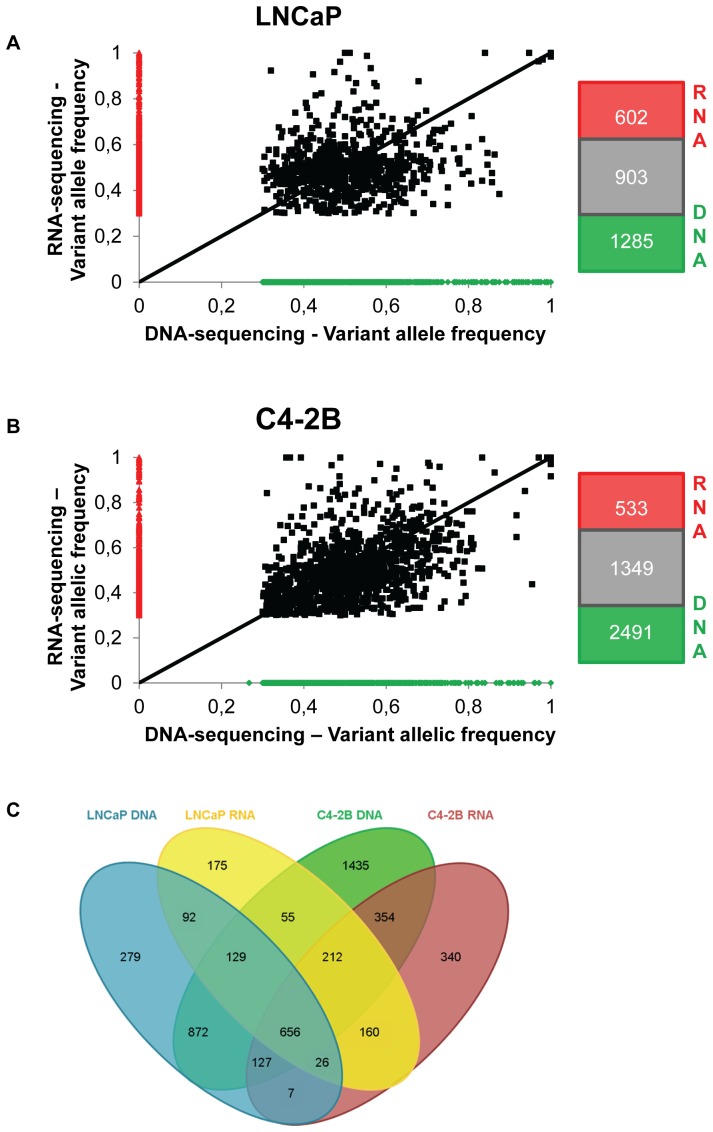
Comparison of variant allelic frequencies measured by whole exome and transcriptome sequencing. **A**–**B**. Comparison of the variant allelic frequency of mutations detected using whole exome and transcriptome sequencing. Black dots are mutations that have been found by both exome sequencing and transcriptome sequencing; red dots were only detected by exome sequencing and green dots only by RNA sequencing. For variant calling, a cut-off of 30% variant allelic frequency was applied. Next to the graph a figure shows the number of mutations from exome sequencing, transcriptome sequencing and the number found by both methods. Graphs are shown for LNCaP (A) and C4-2B cells (B) respectively. **C**. Overlap of all mutations observed by exome and transcriptome sequencing in LNCaP and C4-2B cells.

A comparison of the allele-specific read counts from genome and transcriptome sequencing data of all detected point mutations can be used as a measure of the sequencing quality. The majority of mutations have a similar allele frequency in both DNA and RNA sequencing (Figure 1A–B). Even the few homozygous mutations with allele frequency close to 1 in the exome data, have a similar allele frequency in the RNA sequencing data.

The combination of both the exome and transcriptome sequencing resulted in a total of 2244 mutations common to both cell lines (Figure 1C). Moreover, the number of LNCaP-specific mutations (546) is much lower than that of C4-2B-specific changes (2129), again indicating that mutations have accumulated during the progression to C4-2B. RNA-sequencing confirmed only 41 and 35% of the exonic variants identified by whole exome sequencing of LNCaP and C4-2B. This number rose to 52% when we only took the expressed genes into account (FPKM>1). Conversely, 60 and 71% of the LNCaP and C4-2B variants identified by transcriptome sequencing respectively were confirmed by exome sequencing.

### Nucleotide substitutions

**Figure 2:**
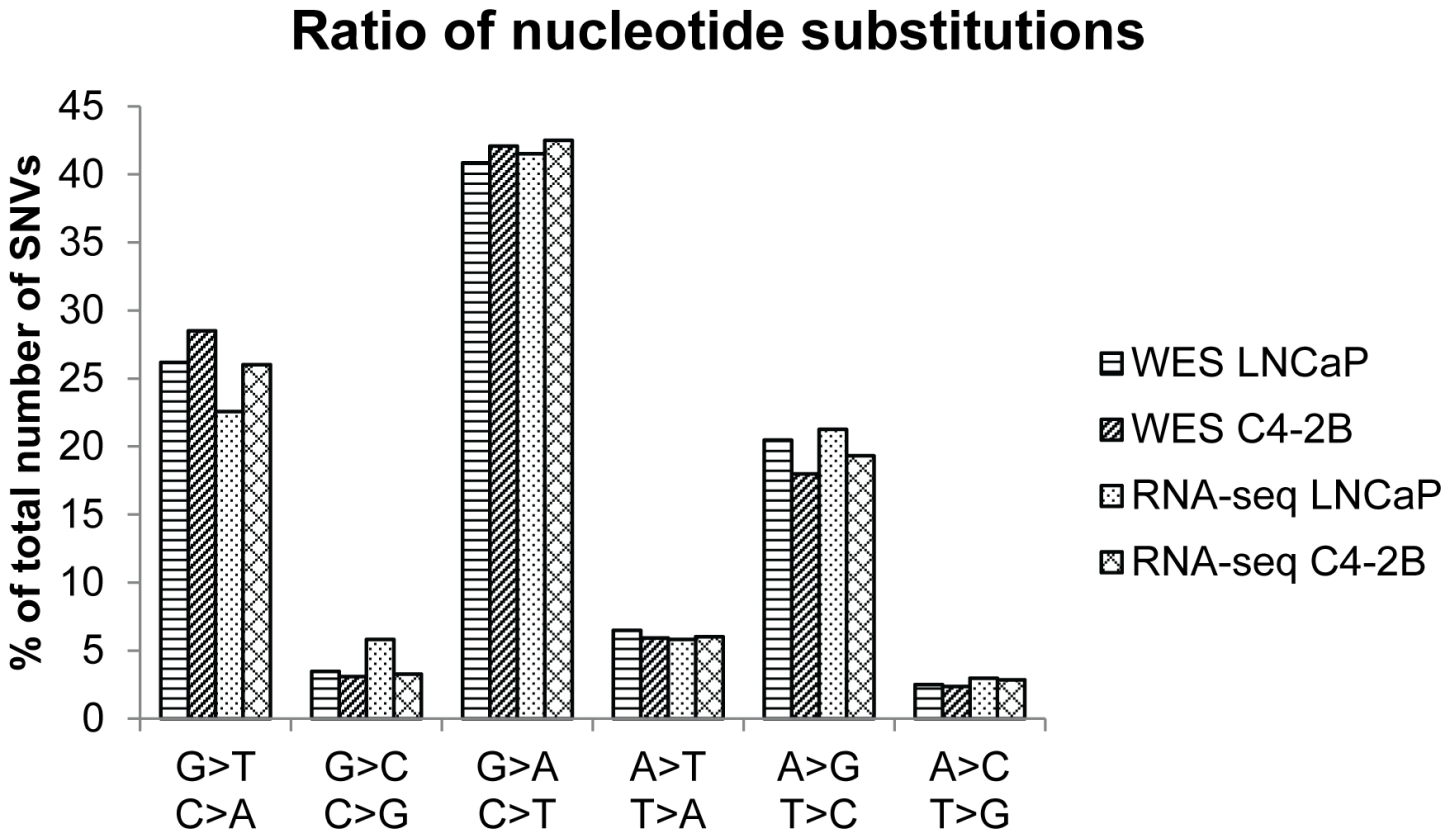
Mutation spectrum of single nucleotide substitutions. Percentages of mutations in each of the six possible mutation classes are represented for the exome sequencing and transcriptome sequencing data of both LNCaP and C4-2B cells.

The different types of transitions and transversions in the exomes and transcriptomes of LNCaP and C4-2B cell lines might give insight in the mutational processes that took place during the development of these cells. We observed that the predominant mutations (40–42%) in both cell lines were G-to-A and C-to-T transitions (Figure 2).

The most prevalent type of RNA editing in higher eukaryotes is the conversion of adenosine to inosine. As inosine is read as a guanine after sequencing, this editing type manifests itself in RNA-sequencing as an A-to-G substitution [18]. However, in our data sets, the number of A-to-G transitions in the exome and the transcriptome sequencing data is comparable arguing against an important role of RNA editing (Figure 2).

### Validation of point mutations

**Figure 3:**
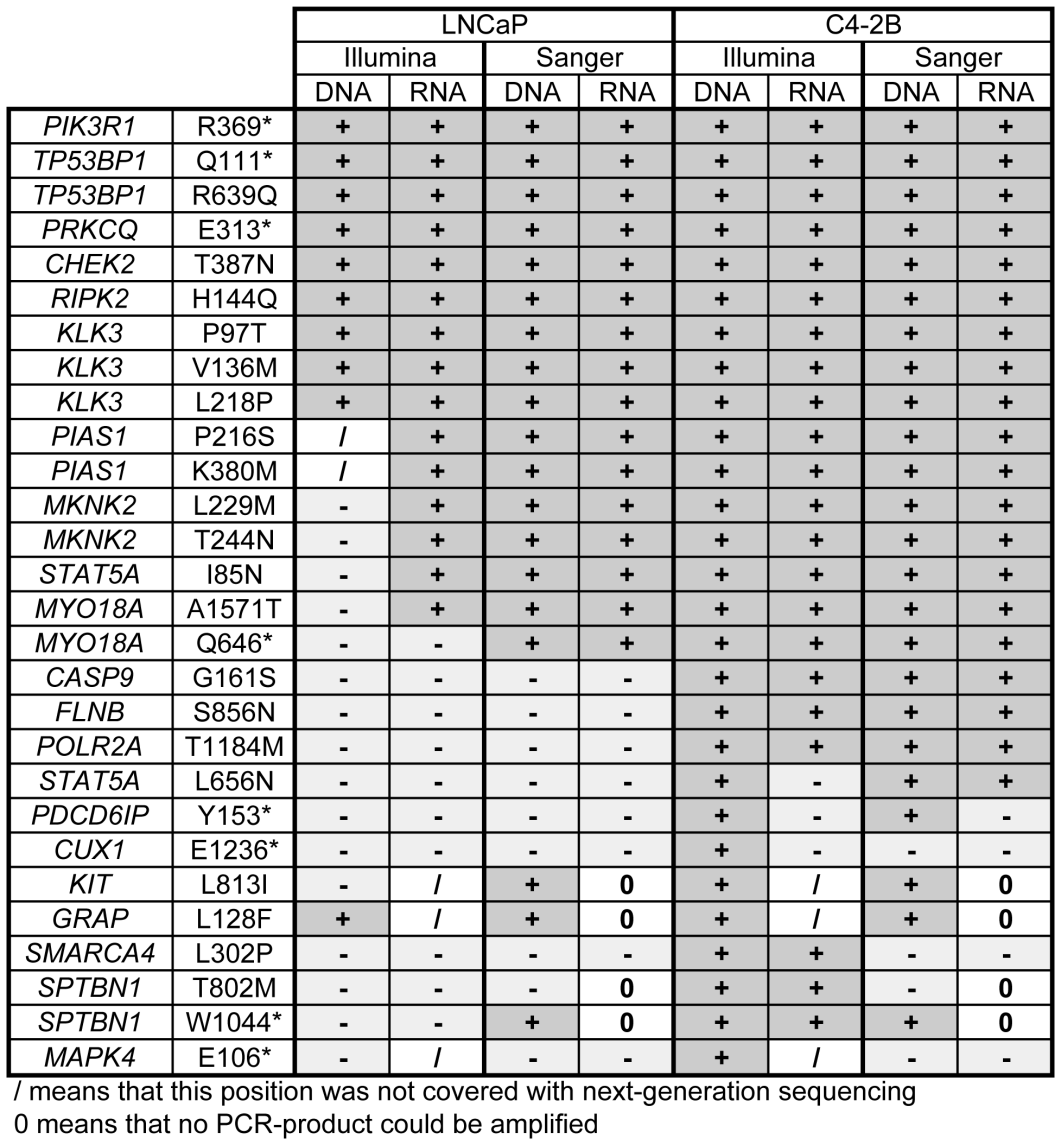
Validation of point mutations in the LNCaP and C4-2B cell lines. Validation was performed using PCR on both genomic DNA and copy-DNA, followed by conventional Sanger sequencing. The first and second column represents the name of the gene and the amino acid substitution respectively. The third, fourth, seventh and eighth column represent next-generation sequencing results for whole exome and transcriptome sequencing, which are then validated with Sanger sequencing in the fifth, sixth, ninth and tenth column. A + denotes that the mutation was detected, a – denotes that the mutation was not detected, while 0 means that no PCR-product could be amplified and/that this position was not covered with next-generation sequencing.

In total, 80 mutations in the exome data from LNCaP (47) and C4-2B (33) were validated by manual Sanger re-sequencing (Figure 3). The genes that were chosen for validation were ranked high in a functional prioritization of all mutated genes in the C4-2B cell line (calculated as in [12]). Nine of these mutations (in PIK3R1, TP53BP1, PRKCQ, CHEK2, RIPK2 and KLK3) were detected by DNA and RNA sequencing in both cell lines, and these were confirmed with Sanger sequencing on genomic and complementary DNA of LNCaP and C4-2B. When we tested seven of the C4-2B exome mutations (PIAS1 P216S and K380M, MKNK2 L229M and T244N, STAT5A I85N and MYO18A A1571T and Q646*), they were not detected by LNCaP exome sequencing, but their presence in the LNCaP genome was evident in the RNA sequencing data and also confirmed by Sanger sequencing on genomic and complementary DNA.

We also detected and confirmed C4-2B specific mutations in CASP9, FLNB, POLR2A and STAT5A in genomic DNA and cDNA of C4-2B cells, but not in LNCaP cells. Finally, mutations in genes that are not expressed in LNCaP or C4-2B (KIT and GRAP) could only be confirmed on genomic DNA.

In conclusion, the GATK UnifiedGenotyper for variant calling which we combined with our extensive filtering generated few false positives. Similar results were shown recently by Liu *et al.* by comparing GATK with SAMtools, Atlas 2 and glftools [19]. Moreover, it should be noted that our validations indicated that the exome analyses did not uncover all mutations, but the variations that were discovered most likely are genuine mutations.

### Differential gene expression between LNCaP and C4-2B cells

We next wanted to search for differentially expressed genes between the two cell lines, since these might provide clues to the mechanisms behind the evolution of LNCaP cells into C4-2B cells. Differential expression was called by the Tuxedo algorithm based on RNA-seq data of triplicates for each cell line, with additional filtering of log2-fold change>2 and q-value<0.001. All replicates were very similar, as can be seen in Figure S3. Moreover, the squared coefficient of variation, which is a normalized measure of cross-replicate variability, is below 0.05 for expressed genes.

**Figure 4:**
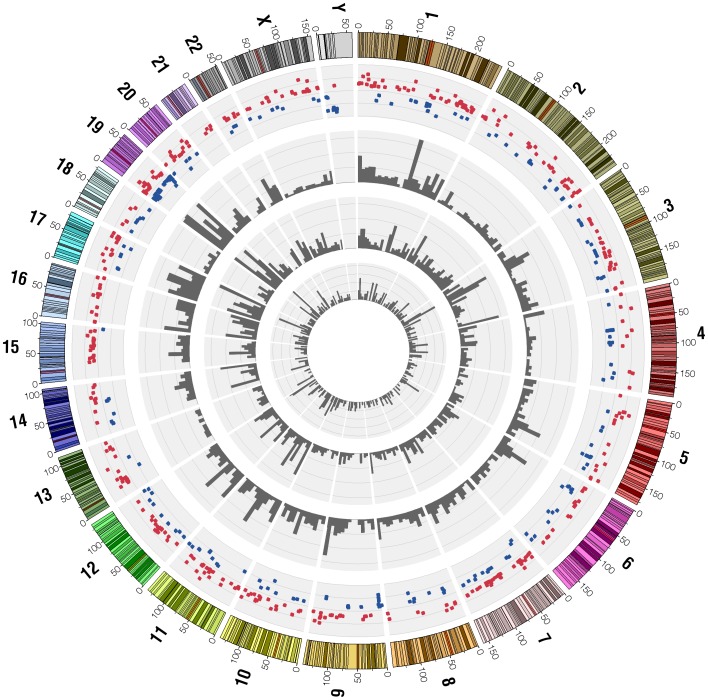
Representation of the catalog of differentially expressed genes and mutations in LNCaP and C4-2B cells. Chromosome ideograms are shown around the outer ring and oriented pter-qter in a clockwise direction with centromeres indicated in red. The outer ring represents differentially expressed genes: 457 genes with higher expression in C4-2B (red dots) and 246 genes with higher expression in LNCaP (blue dots). Other tracks contain (from outside to inside): 2244 mutations in common between LNCaP and C4-2B cells, 2129 mutations specific for C4-2B cells and 546 mutations specific for LNCaP cells shown by density per 10 megabases.

Our analysis resulted in the identification of 703 differentially expressed genes (Table S6), of which 457 genes are higher expressed in C4-2B and 246 are higher expressed in LNCaP cells (Figure S2). An overview of the location of differentially expressed genes across the genome can be found in Figure 4, together with the frequency of point mutations detected in both cell lines, in C4-2B cells only, or in LNCaP cells only. Quantitative RT-PCR on five differentially expressed genes confirmed the RNA-seq data (data not shown).

Fu *et al.* already described some differentially expressed genes between LNCaP and C4-2B, but none of the genes they detected are differentially expressed in our data [20]. We propose that culture conditions and differences between detection platforms most likely explain this discrepancy. On the other hand, there is considerable overlap of our datasets with those of other studies that compared LNCaP and C4-2 transcriptomes [21]–[25].

### Pathway analysis of genomic and transcriptomic data sets

LNCaP and C4-2B cells continue to be used in basic and preclinical research. We propose our databases of mutations and differentially expressed genes as important sources of inspiration for further research projects. In addition, these databases can now be checked for specific mutations before one starts using these cells to study any specific PCa-related pathway.

**Figure 5:**
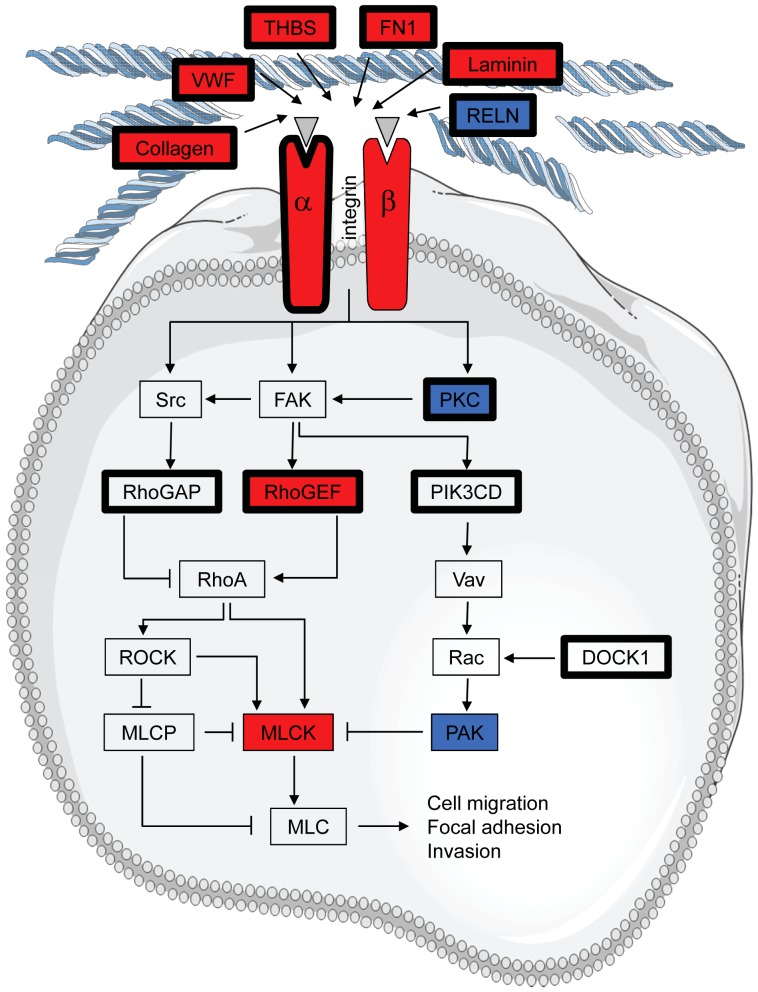
Alterations in the pathway converging on overexpression of Myosin Light Chain Kinase in C4-2B cells. Alterations are defined as having an increased (red) or decreased (blue) expression in C4-2B compared to LNCaP or by somatic mutations in C4-2B cells only (bold black lines). Overexpression of myosin light chain kinase in C4-2B cells might distinguish them from LNCaP cells in cell migration and focal adhesion characteristics.

This paragraph gives an example of a hypothesis based on *in silico* analysis of our data. Pathway-Express analysis of the C4-2B specific mutations combined with the 703 genes differentially expressed between LNCaP and C4-2B cells indicated that the most significant changes were found in the ECM-receptor interaction pathway and in focal adhesion. Both pathways converge in the upregulated expression of the myosin light chain kinase (MLCK) gene (Figure 5).

## Discussion

### A high mutation rate in LNCaP and C4-2B cells

C4-2B cells are derived from a bone metastasis in nude mice inoculated with cells originating from the LNCaP-derived, castration-resistant xenografts called C4-2. They are considered a useful preclinical model for metastatic, castration-resistant and androgen receptor positive PCa. Here, we provide for the first time comparative maps of the point mutations detected in the LNCaP and C4-2B cells. In addition, although transcriptome analyses of LNCaP and C4-2 have been reported, to our knowledge, this is the first transcriptome analysis of C4-2B cells.

C4-2B cells as well as LNCaP cells have a surprisingly high number of point mutations: 4373 and 2790 mutations respectively. Like in primary PCa and castration-resistant PCa samples, the mutational spectrum is dominated by G-to-A and C-to-T transitions [26]–[28]. It is known that mismatch repair defects cause transition mutations, particularly G-to-A and C-to-T substitutions [29]. Hence, most mutations might be caused by the defective mismatch repair system in LNCaP cells, due to the homozygous deletion of the 3′ end of the MSH2 gene [30]. Chen *et al.* already described a correlating high instability of satellite DNA in LNCaP cells [31].

The number of point mutations in our cell lines is much higher than the average 16–33 mutations detected in whole exomes of PCa samples [26], [32]–[34]. These cell lines are therefore atypical, but might be considered a model for cases of PCa in which mismatch repair is defective as described for instance by Barbieri *et al.*, where a single PCa tumor harbored a frameshift mutation of the MSH6 gene among 996 other mutations [32]. Obviously, such higher mutation rates would explain the even higher number of mutations we found in C4-2B compared to LNCaP. Unfortunately, this will also obscure the driver mutations that may have conferred a survival advantage during the metastatic process.

### Link between mutation rates and expression

For both the LNCaP and C4-2B cell line, we see that highly expressed genes more frequently contain point mutations than non-transcribed genes (p<0.0001, Chi Square test, for the highest versus lowest expressed tertile). This contradicts the general link between heterochromatin organization and higher regional mutation rates in human cancer cells [35]. Possibly, in these cell lines, the open chromatin and linked transcription induces more mismatches which normally are efficiently corrected, but not in case of a deficient mismatch repair.

### Comparison of LNCaP and C4-2B mutations

We detected 1784 shared mutations in the exomes of LNCaP and C4-2B, and 2056 C4-2B-specific changes, which makes sense since the C4-2B cells are derived from the LNCaP cells. However, we also detected 404 LNCaP-specific changes, many of which were confirmed by our transcriptome sequences. Obviously, the LNCaP cells we analyzed have deviated from the LNCaP cells that were used originally to develop the C4-2B cells [9]. Indeed, we have shown earlier that even LNCaP cells from different labs are genetically different and while our cells were obtained from ATCC (passage 48), the C4-2B were most likely derived from a much earlier passage of LNCaP cells in 1994 [9], [12].

### Suggestion of a role of MLCK in the metastatic process

Our data can clearly lead to the hypothesis on the metastatic process that took place during the conversion of LNCaP to C4-2B cells. This is exemplified by the convergence of a number of affected pathways to an upregulation of MLCK. Indeed, there are several published links between MLCK and the metastatic process. Discriminant analysis of microarrays identified the MLCK gene as the most informative gene for the PCa genesis process [36], and inhibition of MLCK in rat PCa cells results in reduction of invasiveness, which was principally due to impaired cellular motility [37]. Inhibiting MLCK in fibrosarcoma, pancreatic cancer and breast cancer cells also results in decreased adhesion, migration and invasion and increased apoptosis [38]–[41]. Conversely, activating MLCK leads to an increase in invasion in breast cancer cells and an increased metastatic potential in non-small cell lung cancer [42], [43]. The differential expression of the MLCK gene in the two cell lines investigated here might therefore correlate with the higher metastatic capacity of the C4-2B cells.

## Conclusion

In conclusion, our data clearly show that there are major differences in the number and distribution of mutations and gene expression between LNCaP and C4-2B cells. Since these cell lines are universally used to study the progression from non-metastatic to metastatic PCa, these data are crucial for researchers to correctly interpret their results when using these cell lines. Moreover, our databases will be very helpful in developing new investigational ideas.

## Supporting Information

Figure S1 **FastQC quality control results of the per base qualities.** Output results of the FastQC quality control software (version 0.10.1) are shown here for exome and transcriptome sequencing of LNCaP and C4-2B cells.
(TIF)

Figure S2 **Normalized coverage by position.** The average relative coverage is shown at each relative position along the transcript's length. LNCaP is depicted in green, while C4-2B is depicted in red. The x-axis represents the gene length normalized to 100%, where 0 is the 5′ end of each transcript and 100 is the 3′ end.
(TIF)

Figure S3 **Heatmap of 703 differentially expressed genes.** The heatmap shows the three replicates of each cell line, which are very similar. All differentially expressed genes were detected using the Tuxedo algorithm, with q<0.001 and log2-fold change >2 as cut-offs. It is clear that the majority of genes is upregulated in C4-2B compared to LNCaP, while a smaller group of genes is downregulated in C4-2B.
(TIF)

Table S1 **Sequencing characteristics.**
(XLSX)

Table S2 **Filters used to identify point mutations in the exomes of LNCaP and C4-2B cells.**
(XLSX)

Table S3 **List of point mutations identified in the exome of LNCaP cells.**
(XLSX)

Table S4 **List of point mutations identified in the exome of C4-2B cells.**
(XLSX)

Table S5 **Filters used to identify point mutations in the transcriptomes of LNCaP and C4-2B cells.**
(XLSX)

Table S6 List of 703 genes that are differentially expressed between LNCaP and C4-2B cells.
(XLSX)
