# Supplementary figures and images for: Comparative Genomic and Transcriptomic Analyses of LNCaP and C4-2B Prostate Cancer Cell Lines
Source: PLoS One. 2014 Feb 28;9(2):e90002. doi: 10.1371/journal.pone.0090002 (PMC3938550; doi:10.1371/journal.pone.0090002)

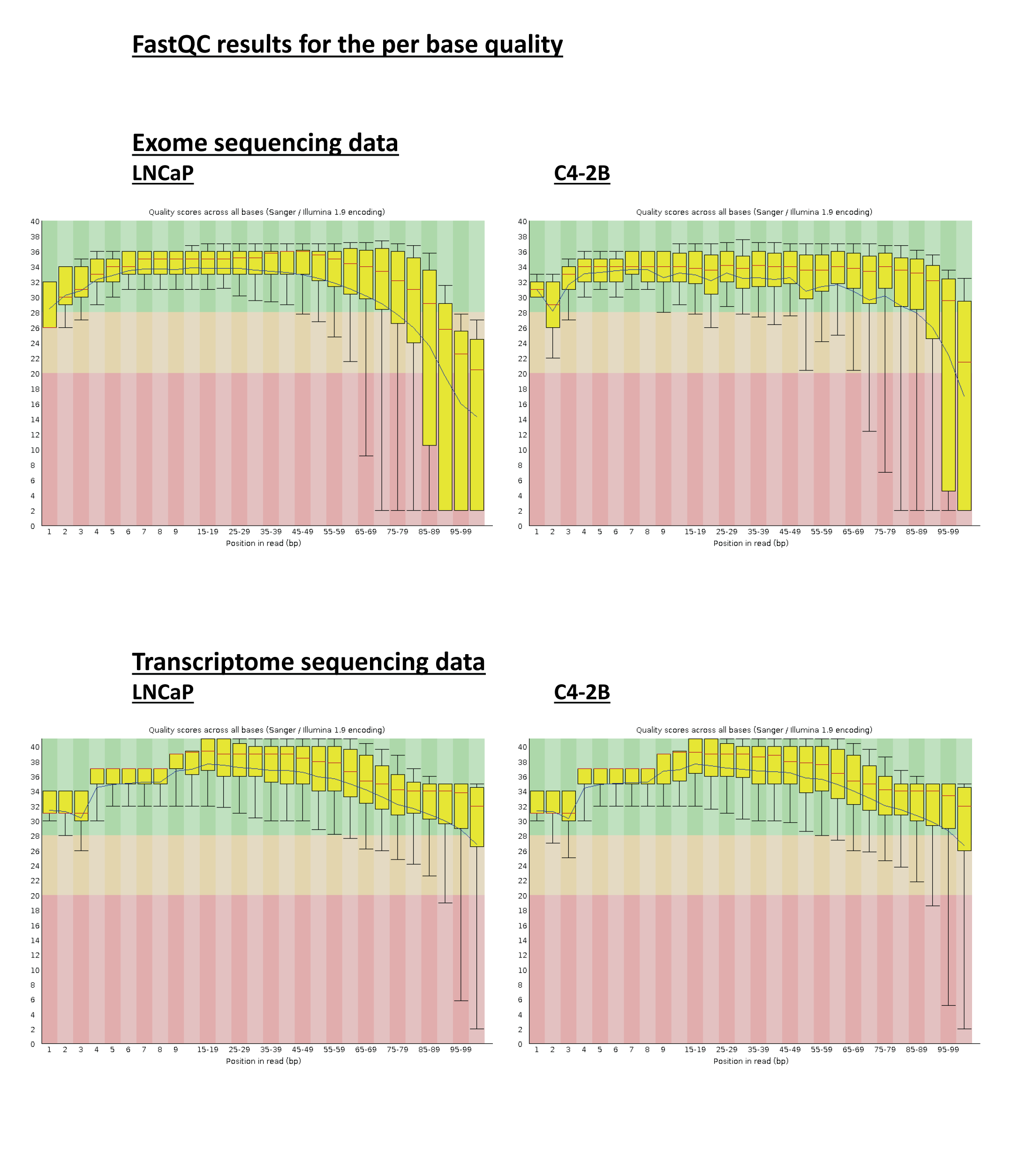

Supplement: Figure S1 — FastQC quality control results of the per base qualities. Output results of the FastQC quality control software (version 0.10.1) are shown here for exome and transcriptome sequencing of LNCaP and C4-2B cells. (TIF) [file pone.0090002.s001.tif]

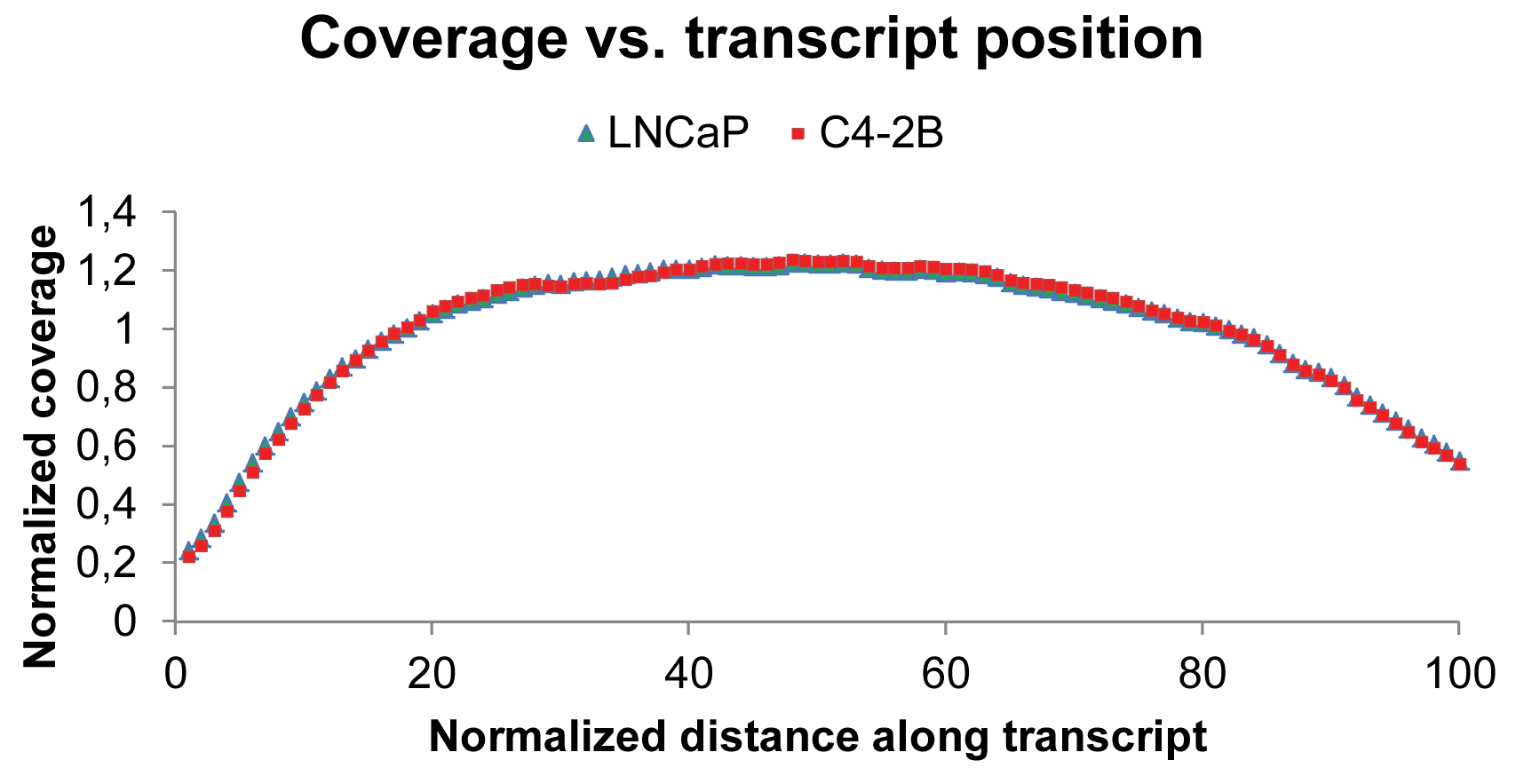

Supplement: Figure S2 — Normalized coverage by position. The average relative coverage is shown at each relative position along the transcript's length. LNCaP is depicted in green, while C4-2B is depicted in red. The x-axis represents the gene length normalized to 100%, where 0 is the 5′ end of each transcript and 100 is the 3′ end. (TIF) [file pone.0090002.s002.tif]

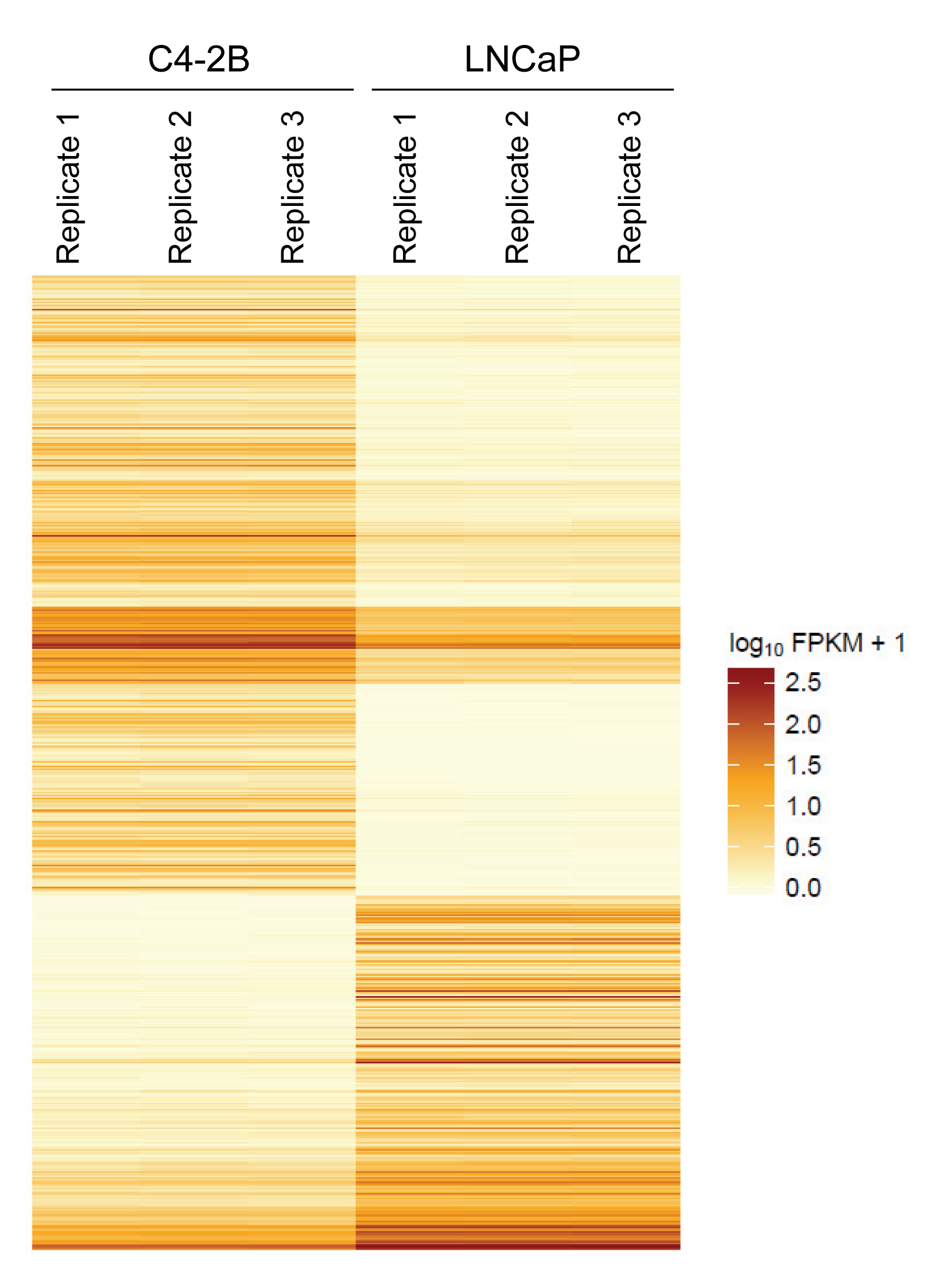

Supplement: Figure S3 — Heatmap of 703 differentially expressed genes. The heatmap shows the three replicates of each cell line, which are very similar. All differentially expressed genes were detected using the Tuxedo algorithm, with q<0.001 and log2-fold change >2 as cut-offs. It is clear that the majority of genes is upregulated in C4-2B compared to LNCaP, while a smaller group of genes is downregulated in C4-2B. (TIF) [file pone.0090002.s003.tif]
